# The liver-derived exosomes stimulate insulin gene expression in pancreatic beta cells under condition of insulin resistance

**DOI:** 10.3389/fendo.2023.1303930

**Published:** 2023-11-07

**Authors:** Azam Mahmoudi-Aznaveh, Gholamreza Tavoosidana, Hossein Najmabadi, Zahra Azizi, Amin Ardestani

**Affiliations:** ^1^ Department of Molecular Medicine, School of Advanced Technologies in Medicine, Tehran University of Medical Sciences, Tehran, Iran; ^2^ Genetics Research Center, University of Social Welfare and Rehabilitation Sciences, Tehran, Iran; ^3^ Centre for Biomolecular Interactions Bremen, University of Bremen, Bremen, Germany

**Keywords:** type 2 diabetes, pancreatic islet, beta cell, liver, organ cross-talk, exosome

## Abstract

**Introduction:**

An insufficient functional beta cell mass is a core pathological hallmark of type 2 diabetes (T2D). Despite the availability of several effective pharmaceuticals for diabetes management, there is an urgent need for novel medications to protect pancreatic beta cells under diabetic conditions. Integrative organ cross-communication controls the energy balance and glucose homeostasis. The liver and pancreatic islets have dynamic cross-communications where the liver can trigger a compensatory beta cell mass expansion and enhanced hormonal secretion in insulin-resistant conditions. However, the indispensable element(s) that foster beta cell proliferation and insulin secretion have yet to be completely identified. Exosomes are important extracellular vehicles (EVs) released by most cell types that transfer biological signal(s), including metabolic messengers such as miRNA and peptides, between cells and organs.

**Methods:**

We investigated whether beta cells can take up liver-derived exosomes and examined their impact on beta cell functional genes and insulin expression. Exosomes isolated from human liver HepG2 cells were characterized using various methods, including Transmission Electron Microscopy (TEM), dynamic light scattering (DLS), and Western blot analysis of exosomal markers. Exosome labeling and cell uptake were assessed using CM-Dil dye. The effect of liver cell-derived exosomes on Min6 beta cells was determined through gene expression analyses of beta cell markers and insulin using qPCR, as well as Akt signaling using Western blotting.

**Results:**

Treatment of Min6 beta cells with exosomes isolated from human liver HepG2 cells treated with insulin receptor antagonist S961 significantly increased the expression of beta cell markers Pdx1, NeuroD1, and Ins1 compared to the exosomes isolated from untreated cells. In line with this, the activity of AKT kinase, an integral component of the insulin receptor pathway, is elevated in pancreatic beta cells, as represented by an increase in AKT’s downstream substrate, FoxO1 phosphorylation.

**Discussions:**

This study suggests that liver-derived exosomes may carry a specific molecular cargo that can affect insulin expression in pancreatic beta cells, ultimately affecting glucose homeostasis.

## 1 Introduction

Diabetes, a metabolic disorder characterized by hyperglycemia, is a growing global health concern affecting millions worldwide. Its two primary forms, type 1 and type 2 diabetes, differ in cause and etiology, but both share a common pathologic feature: a loss of functional beta cell mass characterized by impaired insulin secretion (1). Insulin, a 51 amino acids hormone produced and released by pancreatic beta cells, plays an important role in regulating glucose homeostasis (2). It promotes glucose uptake by the peripheral tissues such as fat and muscle and blocks hepatic glucose production. Defects in insulin secretion can lead to diabetes, underscoring the importance of understanding the complex mechanisms that regulate this critical process (1, 3). Despite the availability of several effective pharmaceuticals for diabetes management, there is a need for novel therapies to safeguard and regenerate pancreatic beta cells (1, 4).

Organisms require communication mechanisms between individual tissues to coordinate their metabolic responses to environmental changes. Communication between the organs enables adaptive responses and adjusted regulation. The liver, muscle, adipose tissue, pancreas, digestive system, and brain all play crucial roles in this dialogue, aiming to efficiently coordinate the mechanisms necessary to maintain glucose homeostasis and energy balance (5). In terms of insulin secretion in response to fluctuations in glucose levels, organ crosstalk is critical for orchestrating a harmonious interplay between tissues, ensuring efficient glucose uptake and utilization (6). Given the pivotal functions of the liver and pancreas in lipid and glucose metabolism, an organism’s ability to adapt to changing nutrient conditions heavily depends on this interaction. Organ communication is facilitated by biological substances released by tissues, which enter the bloodstream and travel to other organs to promote communication. These elements include myokines, adipokines, hepatokines, and extracellular vehicles (EVs) (5, 7–9). For example, the liver also plays a role in regulating the secretion rate and function of islet cells. Hepatocyte growth factors can promote the regeneration of beta cells through c-Met receptors in cases of STZ injection or partial pancreatectomy (10). SerpinB1, secreted by liver cells, regulates the replication of beta cells in mice, and human islets (11).

Exosomes are small EVs with diameters ranging from 30 to 150 nm, and they are secreted by various cell types, including liver cells (12, 13). In recent years, exosomes have emerged as key players in intercellular communication, offering significant potential for understanding the complexities of diabetes pathogenesis (14). These nanosized vesicles contain proteins, lipids, and nucleic acids, allowing them to serve as critical mediators in cellular signaling, tissue repair, and disease progression (15).

As researchers continue to uncover the intricacies of exosome biology, its role in inter-organ communication, especially in insulin secretion and glucose regulation, has become the focal point of intensive research (16). Understanding the mechanisms governing exosome release, cargo selection, and their subsequent impacts on insulin-producing pancreatic beta cells could pave the way for novel therapeutic opportunities for diabetes treatment. By harnessing the potential of these small vesicles, it may be feasible to develop targeted interventions that enhance insulin secretion and improve glycemic control, offering hope for more effective diabetes management and the mitigation of related complications (16, 17). In this context, a recent study by Jalabert et al. discovered that exosomes released from skeletal muscle in an insulin-resistance environment can foster beta cell proliferation, primarily mediated by exosomal miR-16 (18). Consistently, liver-derived EVs from high-fat diet (HFD)-induced obese mice potently stimulate beta cell replication. Moreover, hepatocyte-derived exosomes have demonstrated the remarkable ability to enhance insulin sensitivity, both in *in vitro* experiments and in living organisms. In both scenarios, specific miRNAs emerge as pivotal regulators, orchestrating the metabolic impact on recipient cells (19, 20).

Investigating the impact of liver exosomes on insulin secretion represents a cutting-edge approach that may hold promise for future therapeutic interventions in diabetes. Understanding the liver-islet intercellular communication pathways and their regulation could open new avenues for developing targeted treatments to enhance insulin secretion, ultimately leading to improved glycemic control and better quality of life for individuals with diabetes. This study aims to determine whether and how liver-derived exosomes impact pancreatic beta cell function.

## 2 Materials and methods

### 2.1 Cell lines and cell culture

The murine insulin-secreting beta cell line Min6 was provided by the Iranian Biological Resource Center and cultured in DMEM High-Glucose (Biowest, France) containing two mM L-glutamine (Sigma-Aldrich, Germany). The culture medium was supplemented with 15% fetal bovine serum (Biosera, France), 50μM β-mercaptoethanol (Merk, Germany), 20 mM HEPES (Biowest, France) and 1% penicillin/Streptomycin (Gibco, Germany). HepG2 cells, human hepatoma, was kindly provided by Sørge Kelm (University of Bremen, Germany), and were grown in DMEM High-Glucose (Biowest, France) with 10% fetal bovine serum (Biosera, France), and 1% penicillin/Streptomycin (Gibco, Germany). All cells were maintained in a 5% CO2 humidified incubator at 37°C.

### 2.2 Blocking insulin receptor

To block the insulin receptor (InsR) in liver cells, we utilized a selective insulin receptor peptide antagonist called S961. It consists of a 43-amino acid single-chain peptide. Importantly, S961 exhibits greater selectivity for the insulin receptor than the IGF1 receptor. In both *in vitro* and *in vivo* experiments conducted in rats, S961 was found to completely block insulin activity. Consequently, S961 demonstrates a high affinity and selectivity for the InsR and can be used for both *in vivo* and *in vitro* studies (21, 22). To block InsR in HepG2 cells, different concentrations (1nM, 100nM, 1µM) of S961 were added to the free FBS medium for four hours. The cell lysate was then extracted to determine the amount of Akt phosphorylation (pAkt) by western blotting to determine blocking efficiency. The S961 peptide was generously provided by Novo Nordisk Compound Sharing (Maaloev, Denmark) (23).

### 2.3 MTT assay

To determine the S961 impact on HepG2 cell viability, an MTT (Sigma-Aldrich, USA) assay was conducted. The cells were first seeded in 96-well plates at a density of 10,000 cells/well and allowed to attach for 24 h at 37°C. The S961 blocker was then added to the cells and incubated for 24 h. Then, 5 mg/mL MTT solution was added, and the formazan crystals formed were solubilized in DMSO (Sigma-Aldrich, USA). Finally, absorbance was measured at 570 nm using a microplate reader (Bio-Tek Instruments, Inc., Winooski, USA).

### 2.4 Isolation and characterization of HepG2-derived exosome

HepG2 cells were cultured in starved media for 24h. Exosomes were extracted from the conditioned media. Briefly, cell debris and dead cells were eliminated by centrifugation at 2,000 × g for 30 min. The supernatant fraction was filtered through a 0.22 μm filter and concentrated using a 100 kDa Amicon filter (Merck Millipore, Germany). The exosomes were isolated using the Total Exosome Isolation Kit (Invitrogen, USA) according to the manufacturer’s instructions. To obtain exosomes from insulin receptor-blocked HepG2, 1µM of S961 was added at the beginning of starvation. Exosome proteins were quantified using a BCA Protein Quantification Kit (DNAbiotech, Iran), and exosomal markers, including CD9, CD63, and CD81, were measured using western blot analyses. Transmission Electron Microscopy (TEM) assess the morphology and size of exosomes. In brief, a 20μl droplet of isolated exosomes was applied to a 300-mesh carbon-coated TEM grid for 2 min. The samples were then negatively stained with 2% aqueous uranyl acetate for 1 min. Subsequently, the grid was left to air dry and examined using a Zeiss EM10C TEM instrument operating at an accelerating voltage of 100 kV.

### 2.5 Exosome labeling and cell uptake assay

According to the manufacturer’s protocol, exosomes were labeled with CM-Dil dye (Invitrogen, USA). First, 1µM of CM-Dil dye was added to the isolated exosomes and incubated for 10 min at 37°C. The labeled exosomes were passed through a 100kDa filter (Merck Millipore, Germany). The labeled exosomes were incubated overnight with Min6 cells in an FBS-free medium. Min6 cells were washed with PBS and imaged using an Optika fluorescence microscope (Optika, Italy).

### 2.6 Western blot

Western blot analyses were performed to characterize exosomes and to determine the amount of pFoxO1/FoxO1 and pAkt. RIPA buffer was used for cell lysis. Equal amounts of protein (25µg) were obtained using a BCA kit (DNAbiotech, Iran) and denatured at 95°C for 10 minutes. Proteins were separated by electrophoresis on a 12% SDS-polyacrylamide gel. The resolved proteins were deposited onto a PVDF membrane and blocked by incubation in a 5% blocking buffer containing BSA (Sigma, Germany). Blots were incubated with primary antibodies against Actin (sc-47778, Santa Cruz Biotechnologies, USA), CD9 (sc-13118, Santa Cruz Biotechnologies, USA), CD63 (sc-5275, Santa Cruz Biotechnologies, USA), CD81(sc-166029, Santa Cruz Biotechnologies, USA), pAkt-S473 (Catalog #9271; Cell Signaling Technology, USA), pFoxO1-Thr24 (Catalog #2599; Cell Signaling Technology, USA) and FoxO1 (sc-374427, Santa Cruz Biotechnologies, USA) at 4°C overnight, followed by three washes in 1X Tris-buffered saline with 0.1% Tween-20 (TBST) buffer, and incubation with horseradish peroxidase (HRP)- conjugated secondary antibodies (sc-516102, Santa Cruz Biotechnologies, USA). After the unbound antibodies were removed by washing with 1X TBST buffer (3X -10 min), the signal was detected using Chemiluminescent HRP Substrate (Millipore, USA).

### 2.7 RNA extraction and qPCR analysis

Total RNA was isolated using the manual phenol-chloroform method, and quantified using an ND-1000 spectrophotometer (Nanodrop Technologies, USA). A total of 500 ng of RNA was used for cDNA synthesis using a cDNA synthesis kit (Applied Biosystems, USA), and random hexamers were used for cDNA synthesis. One microliter of cDNA was used as a template for PCR without dilution, using the Corbett Rotor-Gene Q 6000 real-time PCR detection system (Qiagen Corbett, Germany) in a total reaction volume of 25μl volume reaction. The mixture included 12.5μl of SYBR Green master mix (Amplicon, Denmark) and 10.5μl DW containing the specific forward and reverse primer (**Supplementary Table 1**). The thermal cycler program was performed in triplicate as follows:15 min at 95°C; 40 cycles of 20 s at 95°C, 30 s at 57°C, and 30 s at 72°C, followed by melting curve detection. Relative mRNA quantification was conducted using the comparative 2^−ΔΔCt^ method, with cyclophilin as the reference gene.

### 2.8 Statistical analysis

One-way analysis of variance (ANOVA) was used to assess the statistical differences between the groups under an unequal variance assumption. The mean and standard deviation (SD) of the results were reported. GraphPad Prism Version 9.0 was used to conduct all statistical analyses.

## 3 Results and discussion

**Figure 1:**
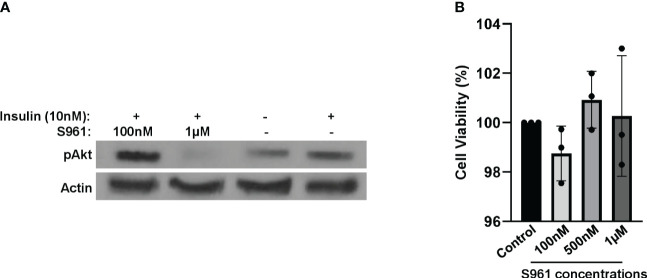
Impact of S961 on insulin receptor signaling and cell survival. **(A)** HepG2 cells were treated with various concentrations of the insulin receptor antagonist S961 for 2h. pAkt and Actin were analyzed by Western Blotting. **(B)** HepG2 cells were treated with different concentrations of the insulin receptor antagonist S961 for 24h at 37°C, and cell viability was assessed by the MTT assay. Data are presented as mean ± SD (n=3). Statistical analysis was performed using one-way ANOVA.

To mimic an insulin-resistant condition *in vitro*, we employed HepG2 cells treated with insulin receptor antagonist S961. Following a two-hour exposure to 1µM of S961, we observed a substantial reduction in insulin-induced Akt activity, as represented by the decrease phosphorylation of Akt at the S473 site- a critical event downstream of the InsR signaling cascade. This effect was notably more pronounced when compared to lower concentrations of S961 and a control group (**Figure 1A**). These findings demonstrate the inhibitory efficacy of S961 on the insulin receptor, consequently leading to the downregulation of pAkt, a key intermediary in the insulin signaling pathway in liver HepG2 cells. It has been documented that IGF-1R exhibits a substantial expression within HepG2 cells, exerting a significant influence on cell proliferation, migration, and anti-apoptosis through the activation of PI3K-Akt and ERK signaling pathways (24, 25). It is important to note that insulin possesses the capability to activate IGF-1R with a notably lower affinity compared to InsR. Furthermore, as mentioned above, S961 can also block IGF-1R, albeit with reduced affinity compared to InsR. Consequently, the potential S961-induced inhibition of IGF-1R raises the intriguing possibility of altering the composition and secretion of extracellular vesicles, such as exosomes, in our context. Additional mechanistic investigations are required to disclose this possibility. In order to confirm that the concentrations of S961 used in our experiment did not exert adverse effects on cell viability, we conducted an MTT assay. The data revealed no significant toxicity associated with the concentrations of S961 used (100nM, 500nM, and 1µM) when HepG2 cells were exposed for 24 hours (**Figure 1B**).

**Figure 2:**
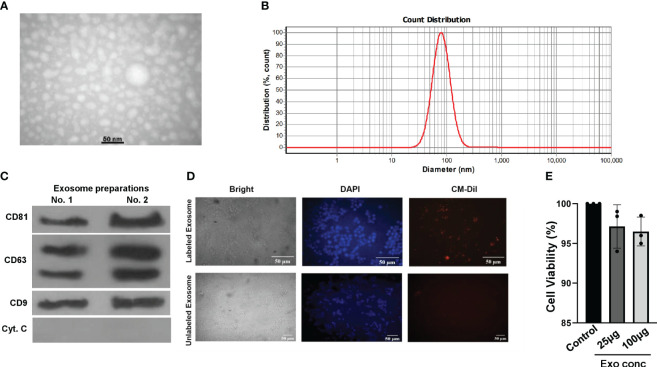
Characterization and uptake of HepG2 cell-derived exosomes. **(A)** TEM image of purified HepG2 cell-derived exosomes. **(B)** DLS analysis yielded the nanoparticle size distribution for HepG2 exosomes. **(C)** Western blot analysis of exosomal markers CD63, CD9, CD81, and cell-specific marker Cytochrome C in exosomes isolated from HepG2 cells. Both independent isolations are shown. **(D)** Microscopy images of CM-Dil-labeled HepG2-exosomes inside Min6 cells. Min6 cells were incubated with CM-Dil dye-labeled exosomes for 24h, and cells were incubated with labeled exosomes and unlabeled ones as a negative control. **(E)** Min6 beta cells were treated with different concentrations of exosomes isolated from HepG2 cells for 24 hours at 37°C, and cell viability was assessed using the MTT assay. Data are presented as mean ± SD (n=3). Statistical analysis was performed using one-way ANOVA.

Next, we proceeded to isolate exosomes from HepG2 cells, both in the presence and absence of S961. To ensure the purity and size of these exosomes, we employed a complementary set of validation methods. Initially, transition electron microscopy (TEM) analyses provided visual confirmation of the isolated particles, revealing the presence of typical exosomal characteristics (**Figure 2A**). Further assessment using dynamic light scattering (DLS) confirmed the particle size distribution of HepG2 exosomes, indicating peak particle sizes of approximately 80.1 nm, which aligns with the expected size range for exosomes (**Figure 2B**). Finally, Western blot analysis solidified our findings by demonstrating an enrichment exosomal markers, specifically CD63, CD9, and CD81. Notably, the cell-specific marker cytochrome C was absent in the isolated exosomes (**Figure 2C**). These combined results collectively confirm the purity and distinctive features of the isolated exosomes.

To investigate the potential uptake and internalization of exosomes by Min6 beta cells, we employed a method involving the fluorescent labeling of exosomes from HepG2 cells with CM-Dil. These labeled exosomes were then incubated with Min6 cells for a 24-hour period, and we subsequently examined their intracellular localization using fluorescence microscopy (**Figure 2D**). The results revealed that CM-Dil-labeled exosomes were indeed internalized by Min6 cells, appearing as endosome-like vesicles within the cytoplasm. These results strongly indicate the capability of Min6 cells to actively take up and internalize exosomes produced by HepG2 cells, thereby confirming the preserved structural integrity of the isolated exosomes. Furthermore, in order to assess any potential cytotoxic effects, we conducted an MTT assay using varying concentrations of exosomes isolated from HepG2 cells. Notably, the results demonstrated that at concentrations of 25 and 100 µg/ml of exosomes, there was no observed toxicity in Min6 cells (**Figure 2E**). These findings underscore the safety and biocompatibility of the exosomes, further supporting their suitability for subsequent investigations.

**Figure 3:**
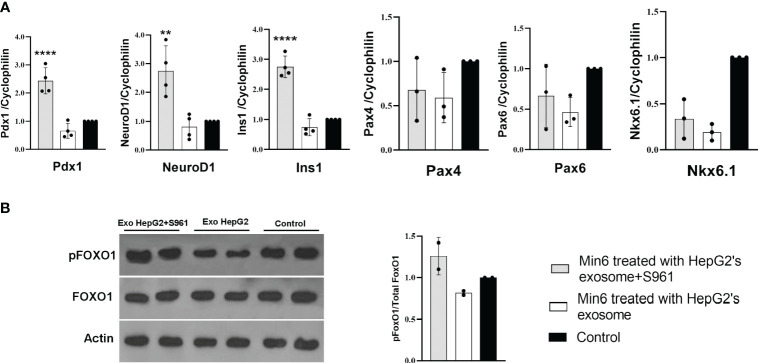
Influence of HepG2 derived exosomes on beta cell markers. Min6 beta cells were treated with exosomes isolated from HepG2 in the presence or absence of S961 for 24h. **(A)** qPCR for Pdx1, NeuroD1, Ins1, Nkx6.1, Pax6, and Pax4 mRNA expression in Min6 cells normalized to cyclophilin. Data are presented as mean ± SD (n=3-4). **(B)** Representative Western blot and pooled quantitative densitometry analysis of pFOXO1, FOXO1 and Actin in Min6 cells (n=2). Statistical analysis was performed using one-way ANOVA. **p < 0.01; ****p < 0.0001.

To investigate the impact of HepG2-derived exosomes, isolated under control or insulin-resistance condition, on pancreatic beta cell function, we assessed the expression of critical transcription factors involved in insulin gene regulation, including Pdx1 and NeuroD1 (26). Min6 beta cells were treated with exosome isolated from HepG2 cells exposed to S961 at a concentration of 100µg/ml, resulting in a significant increase in the mRNA expression of Pdx1 and NeuroD1 compared to control exosomes (**Figure 3A**). Consequently, the expression of insulin (Ins1) was upregulated by exosomes obtained from S961-tretaed cells. In contrast to Pdx1 and NeuroD1, the gene expression of beta cell markers, namely Pax4, Pax6, and Nkx6.1, remained unaltered in response to S961-treated exosomes (**Figure 3A**). These results underscore a notable enhancement in the expression of Pdx1 and NeuroD1, ultimately leading to increased Ins1 expression.

Pdx1 plays a crucial role pancreatic and islet endocrine development, as well as in beta cell function and viability in mammals (27). Studies have shown that increased Pdx1 expression not only enhances the proliferation and differentiation of beta cells but also elevates insulin levels and stimulates glucose-induced insulin transcription (26, 28). Importantly, the orchestration of Pdx1 transcription is a multifaceted process, requiring specific enhancer elements. Transcription factor Foxa2 plays a pivotal role, as it directly binds to PDX1 enhancer elements during development. The genetic deletion of Foxa1 and Foxa2 within the PDX1 lineage has profound implications, leading to pancreas agenesis, underscoring the crucial early role of Foxa1 and Foxa2 in governing PDX1 expression and, consequently, the expansion and differentiation of pancreatic endocrine cells (29). Importantly, the interconnected and cooperative actions of Foxa2 and PDX1 also extend to the postnatal maturation of pancreatic beta cells (30).

Similar to Pdx1, the NeuroD1 gene is necessary for islet cell development and beta cell function (31). Previous research has revealed that several transcription factors collaborate to control the expression of the Ins gene, and no single factor can independently induce ins gene expression on its own (32). Both Pdx1 and NeuroD1 contribute to regulation of insulin expression (33). This could explain the simultaneous increase in these important transcription factors, Pdx1 and NeuroD1, which facilitates an efficient induction of insulin gene expression. Consistent with our findings, a previous study indicated that an increase in the expression of these two genes was associated with an increase in insulin levels (34). Ohneda et al. demonstrated that Pdx1 interacts with NeuroD1 and other transcription factors within the DNA promoter region of the insulin gene. This interaction results in the formation of an active transcription activation complex through protein-protein interactions, providing multiple platforms for communication with other transcriptional activators to finely regulate insulin gene expression (35).

Notably, Nkx6.1, a transcription factor intricately involved in the growth and function of pancreatic beta cells (36), along with Pax4 and Pax6, transcription factors essential for shaping pancreatic endocrine development (37), may not have a direct impact on the enhancement of insulin gene expression. These observation is in line with our data, which demonstrates that exosomes treated with S961 fail to induce these specific genes. This data strongly suggests that Nkx6.1, Pax4, and Pax6 play a non-essential role in the upregulation of the Ins1 gene within the context of our experimental setting.

Protein phosphorylation is important component of intracellular signaling pathways, playing a vital function in signal transduction that ultimately determines the fate of cells (38, 39). AKT kinase stands as the key pro-survival kinase in beta cells. The activation of a signaling pathways downstream of mitogen receptors, such as insulin, insulin-like growth factors (IGF family), and phosphoinositide 3-kinase (PI3K) (40, 41) is pivotal in regulating beta cell proliferation, apoptosis, and insulin secretion. AKT-mediated phosphorylation of multiple substrates, including the Forkhead transcription factor FoxO family, positively regulates insulin transcription, insulin secretion, and the growth and survival of beta cells (42–44) Notably, treating Min6 cells with exosomes isolated from HepG2 cells exposed to S961 led to a moderate increase in the phosphorylation of Akt kinase substrate FoxO1 indicating higher Akt activity (**Figure 3B**). FoxO1 regulates glucose levels, cell cycle progression, and apoptosis. When FoxO1 is phosphorylated, it is unable to enter the nucleus, reducing its capacity to regulate gene expression associated with stress responses, metabolic functions, and apoptosis. Instead, phosphorylated FoxO1 promotes cell survival and growth (45, 46). Elevating pFoxO1 levels and consequently inhibiting it could enhance insulin secretion capacity by positively influencing beta cell survival, glucose metabolism, insulin gene expression, and exocytosis. In our study, the amount of pFoxO1 was found to be positively correlated with the expression of Ins1. This phenomenon was elucidated in a study conducted by Kawamori et al. (47). pFoxO1 retains Pdx1 in the cell nucleus, potentially leading to an increase in Ins1 expression. In line with this, when the activity of the PI3K/Akt/FoxO1/Pdx1 signaling pathway intensifies in the islets, Pdx1 translocates from the cytoplasm to the nucleus, resulting in improved insulin secretion and thereby mitigating the effects of hyperglycemia (48).

## 4 Conclusion

In conclusion, our study indicated that insufficient insulin signaling in liver cells could trigger the release of exosomes, which may stimulate insulin gene expression and potentially insulin secretion in beta cells. Exosome treatment led to significant upregulation of Pdx1, NeuroD1, and Ins1 expression, suggesting an enhancement of beta cell function and insulin production. Nonetheless, to conclusively establish whether the rise in insulin gene expression mediated by liver-derived exosomes can translate into enhanced glucose-stimulated insulin secretion, we recognize the need for additional mechanistic experiments on primary islet/beta cells. An additional aspect worth noting is the potential for distinct interplay between species, given that Min6 cells are of mouse origin while HepG2 cells are human. While essential mediators released from metabolic organs, like the liver, often exhibit a degree of conservation across different species such as SerpinB1, a liver-derived secretory protein known to regulate beta cell proliferation in humans, mice, and zebrafish (11), there remains the possibility that variations in exosome components and their consequential effects may arise due to inter-species differences.

These findings have promising implications for future research on diabetes therapeutics. Enhancing insulin secretion and recovering beta cell function in individuals with beta cell dysfunction can be achieved through the imperative and effective utilization of exosomes as a delivery system to elevate the levels of glucose-induced insulin secretion. Additionally, investigating the specific contents of these exosomes could offer valuable insights into the molecular mechanisms underlying their effects on beta cell transcription factors. While our study focused on the effects of exosomes on Min6 cells, future studies could expand this research to include *in vivo* models or primary human beta cells. Our findings contribute to the growing knowledge of exosome-mediated intercellular communication and its potential role in beta cell biology. Harnessing the therapeutic potential of exosomes may pave the way for innovative and targeted treatments of diabetes and related metabolic disorders.

## Data availability statement

The raw data supporting the conclusions of this article will be made available by the authors, without undue reservation.

## Ethics statement

Ethical approval was not required for the studies on humans in accordance with the local legislation and institutional requirements because only commercially available established cell lines were used. Ethical approval was not required for the studies on animals in accordance with the local legislation and institutional requirements because only commercially available established cell lines were used.

## Author contributions

AA: Conceptualization, Funding acquisition, Methodology, Resources, Supervision, Writing – review & editing. AM-A: Formal Analysis, Investigation, Methodology, Writing – original draft. GT: Resources, Writing – review & editing. HN: Resources, Writing – review & editing. ZA: Conceptualization, Funding acquisition, Methodology, Supervision, Writing – review & editing.
