# Supplementary material for: The liver-derived exosomes stimulate insulin gene expression in pancreatic beta cells under condition of insulin resistance
Source: Front Endocrinol (Lausanne). 2023 Nov 7;14:1303930. doi: 10.3389/fendo.2023.1303930 (PMC10661932; doi:10.3389/fendo.2023.1303930)
Supplement: Supplementary file 1 [file Table_1.docx]

**Supplementary Table 1.** The primers used in this study

| **Primer name** | **Sequence 5'→ 3'** | **Product length** |
| --- | --- | --- |
| Pdx1-F | ATGAAATCCACCAAAGCTCAC | 131bp |
| Pdx1-R | AATTCCTTCTCCAGCTCCAG |  |
| NeuroD1-F | AGATCGTCACTATTCAGAACCT | 167bp |
| NeuroD1-R | GTCCTCTTTCTTGTCTGCCT |  |
| Ins1-F | CCACCCAGGCTTTTGTCAAA | 145bp |
| Ins1-R | CCCAGCTCCAGTTGTTCCAC |  |
| Nkx6.1-F | GTCGCTCGTCTCACCTCAC | 130bp |
| Nkx6.1-R | TGCCACGCTTTTTCAAGACG |  |
| Pax4-F | CACCAGGCAGATGTTCCAG | 173bp |
| Pax4-R | GGTTGATGGCACTTGTCCT |  |
| Pax6-F | TGAATGGGCGGAGTTATGAT | 126bp |
| Pax6-R | GGACGGGAACTGACACTC |  |
| Ppia-F | CAAGACTGAATGGCTGGATG | 130bp |
| Ppia-R | GTCGGAAATGGTGATCTTCT |  |
